# Lack of paternal silencing and ecotype-specific expression in head and body lice hybrids

**DOI:** 10.1093/evlett/qrae003

**Published:** 2024-02-06

**Authors:** Hollie Marshall, Andrés G de la Filia, Ross Cavalieri, Eamonn B Mallon, John M Clark, Laura Ross

**Affiliations:** School of Biological Sciences, Institute of Evolutionary Biology, The University of Edinburgh, Edinburgh, United Kingdom; The Department of Genetics and Genome Biology, University of Leicester, Leicester, United Kingdom; School of Biological Sciences, Institute of Evolutionary Biology, The University of Edinburgh, Edinburgh, United Kingdom; Massachusetts Pesticide Analysis Lab, Veterinary and Animal Sciences, University of Massachusetts Amherst, Massachusetts, United States; The Department of Genetics and Genome Biology, University of Leicester, Leicester, United Kingdom; Massachusetts Pesticide Analysis Lab, Veterinary and Animal Sciences, University of Massachusetts Amherst, Massachusetts, United States; School of Biological Sciences, Institute of Evolutionary Biology, The University of Edinburgh, Edinburgh, United Kingdom

**Keywords:** *Pediculus humanus*, genomic imprinting, paternal genome elimination, alternative splicing, DNA methylation

## Abstract

Paternal genome elimination (PGE) is a non-Mendelian inheritance system, described in numerous arthropod species, in which males develop from fertilized eggs, but their paternally inherited chromosomes are eliminated before or during spermatogenesis. Therefore, PGE males only transmit their maternally inherited set of chromosomes to their offspring. In addition to the elimination of paternal chromosomes, diverse PGE species have also repeatedly evolved the transcriptional silencing of the paternal genome, making males effectively haploid. However, it is unclear if this paternal chromosome silencing is mechanistically linked to the chromosome elimination or has evolved at a later stage, and if so, what drives the haploidization of males under PGE. In order to understand these questions, here we study the human louse, *Pediculus humanus*, which represents an ideal model system, as it appears to be the only instance of PGE where males eliminate, but not silence their paternal chromosomes, although the latter remains to be shown conclusively. In this study, we analyzed parent-of-origin allele-specific expression patterns in male offspring of crosses between head and body lice ecotypes. We show that hybrid adult males of *P. humanus* display biparental gene expression, which constitutes the first case of a species with PGE in which genetic activity of paternal chromosomes in the soma is not affected by embryonic silencing or (partial or complete) elimination. We did however also identify a small number of maternally biased genes (potentially imprinted genes), which may be involved in the elimination of paternal chromosomes during spermatogenesis. Finally, we have identified genes that show ecotype-specific expression bias. Given the low genetic diversity between ecotypes, this is suggestive for a role of epigenetic processes in ecotype differences.

## Introduction

Many organisms follow Mendelian inheritance where offspring receive a random chromosome set from each parent. However, Mendel’s laws are broken by 1,000s of species where the inheritance of the maternal or paternal chromosomes is biased (Ross et al., 2022). Paternal genome elimination (PGE) is perhaps the most extreme and common example. It is widely distributed across many arthropod species, in which males develop from fertilized eggs, but their paternally inherited chromosomes are eliminated before or during spermatogenesis. Therefore, PGE males only transmit their maternally inherited set of chromosomes to their offspring (Burt & Trivers, 2006; Gardner & Ross, 2014; Herbette & Ross, 2023; Normark, 2003). In PGE species where elimination of paternal chromosomes takes place early in development (embryonic PGE), such as mesostigmatid mites (Nelson-Rees et al., 1980; Norton et al., 1993) or diaspidid scale insects (Nur, 1980; Ross et al., 2010), males become true haploids and therefore only express maternal alleles. In some PGE species where elimination is delayed until spermatogenesis (germline PGE), such as Scolytinae beetles (Brun et al., 1995), neococcid scale insects (Bongiorni & Prantera, 2003; Brown & Nur, 1964; Ross et al., 2010), or booklice (Hodson et al., 2017), paternal chromosomes are retained throughout development, but become tightly condensed during embryogenesis. As a result, paternal chromosomes remain transcriptionally inactive (Berlowitz et al., 1968) and expression of maternal alleles usually predominates. One of the most intriguing aspects of the evolutionary history of PGE is the repeated convergence toward these adaptations to reduce or completely prevent expression of paternal chromosomes in males in the different groups where PGE has independently emerged. However, we still do not understand why males often become functionally haploid under PGE, and if this is a derived state or if such haploidy evolves at the same time as the biased transmission through meiosis. In other words, is it possible for PGE to evolve with just biased transmission through the germline without any somatic effects on the paternal genome in males?

In order to understand how PGE, and the associated loss of paternal gene expression, has evolved, we need to examine species that display potentially ancestral forms, ideally, those with only germline elimination and very limited or no somatic effects. The human louse *Pediculus humanus* is currently the only species that might fit the bill: Male lice eliminate their fathers genome during spermatogenesis and only (or predominantly) transmit maternally inherited alleles to their offspring (de la Filia et al., 2018; McMeniman & Barker, 2006). Similar to other PGE taxa, male lice have a strongly modified spermatogenesis: The first meiotic division is followed by a series of mitosis to form a 32/64 cell cyst; of which, only half (those containing maternal chromosomes) continue to develop as active spermatozoa, while the others degenerate in situ (Bressa et al., 2015; Hindle & Pontecorvo, 1942). However, none of the somatic phenomena associated with PGE—heterochromatization, elimination of single paternal chromosomes or the whole paternal set during early development—have ever been described in human lice or other members of the order Phthiraptera, despite several cytogenetic studies having been conducted (Bressa et al., 2015; Doncaster & Cannon, 1920; Golub & Nokkala, 2004; Tombesi & Papeschi, 1993). Previous genetic studies of PGE in lice (de la Filia et al., 2018; McMeniman & Barker, 2006) focused only on patterns of inheritance, not expression of paternal alleles. It therefore remains unknown as to whether the paternal chromosomes in males are transcriptionally active or not.

From an evolutionary perspective, human lice could represent the most basal form of PGE, where germline elimination occurs without, or with very little reduction in, paternal gene expression. This would make it a promising system for understanding the evolution and mechanism of haploidization of PGE males. The most widely cited hypothesis explaining this phenomenon focuses on reducing genomic conflict between the paternal and maternal genomes in males, where silencing of the paternal genome is thought to be a maternal adaptation to counteract resistance of paternal alleles to germline elimination (Herrick & Seger, 1999; Ross et al., 2010). Under this scenario, a lack of paternal silencing might explain the high rate of paternal allele transmission through males (incomplete PGE) that we have previously found in human body lice (de la Filia et al., 2018). However, this observation is suggestive at most, and there is as yet no convincing empirical evidence supporting the genomic conflict hypothesis. *Pediculus humanus* could therefore be a promising system to directly search for genes or chromosomal regions with parent-of-origin-specific expression that could be directly involved in the induction—or deterrence—of paternal chromosome elimination, an analysis that would be impossible in other taxa with complete silencing of the paternal genome in males. Specifically, imprinted genes involved in either chromosome behaviour during spermatogenesis or involved in epigenetic processes that could underlie the recognition of paternal chromosome, e.g. modifiers of DNA methylation, or histone modifications, would be of particular interest.

Moreover, somatic patterns of gene expression in *P. humanus* are also interesting beyond fundamental theories on intragenomic conflict and evolution of non-canonical genetic systems. Many PGE species with paternal chromosome silencing are pests or parasites that pose a severe economic burden on crop production (Damon, 2000; Gill & Kosztarab, 1997). *Pediculus humanus* is a widespread human ectoparasite with serious consequences (Clark et al., 2013). Head and body lice are two distinct ecotypes of *P. humanus*, differing in morphological and behavioral traits driven by ecological factors (Light et al., 2008; Veracx & Raoult, 2012). Body lice constitute a serious health threat as vectors of severely pathogenic bacteria (Roux & Raoult, 1999), and head lice infestations have been estimated to cause costs of hundreds of million dollars every year (Hansen & O’Haver, 2004). Recently, increasing resistance to available treatments has become a major challenge to human louse control (Burgess, 2004; Clark, 2018; Clark et al., 2015; Durand et al., 2012), creating the need for novel resistance-proof management programs. The overlooked transmission patterns of PGE in both ecotypes could help us better understand how pesticide resistance evolves in lice populations, as haploid versus diploid expression in males will affect the likelihood of spread of recessive resistance alleles, as well as their effects in males and females. Yet, a full characterization of the form of PGE present in *P. humanus* is necessary to determine whether existing models of resistant evolution in haplodiploid taxa (Caprio & Hoy, 1995; Carrière, 2003; Crozier, 1985; Denholm et al., 1998) can beapplied.

In this study, we analyzed parent-of-origin allele-specific expression patterns in F1 male offspring of crosses between head and body lice. The transcriptomic profiles of both ecotypes are highly similar, with low levels of interecotype divergence in nucleotide sequences and gene expression levels (Olds et al., 2012). Therefore, individuals from both ecotypes can be crossed in laboratory conditions, yielding viable and fertile offspring (Busvine, 1978). We use ecotype-specific alleles to identify the parent-of-origin of sequencing reads in the F1 males. Using this parent-of-origin data, we ask (a) is there expression of paternal chromosomes in hybrid males? (b) If there is paternal expression, is it completely unbiased for all alleles? (c) Is the pattern of paternal expression the same for each ecotype? (d) Aside from studying parent-of-origin-specific expression, this analysis can also provide insights into ecotype-specific expression patterns and therefore have consequences for our understanding of the divergences between them. While not our main focus, we therefore also briefly describe and discuss these results.

## Methods

### Experimental populations and interecotype crosses

A series of intraspecific crosses were set up using individuals from the head louse strain SF-HL and the body louse strain Frisco-BL. The SF-HL colony was established in 2002 from head lice collected from ∼20 infested children in Plantation, Miami, and Homestead (FL, USA). Approximately 50 males and 50 females were used to temporarily establish a colony on human volunteers (Takano-lee et al., 2003). Fertile eggs from Homestead were added to the colony at least three times between 2002 and 2006. Approximately 30–50 eggs were introduced each time. The colony was placed on an in vitro rearing system in 2006 (Yoon et al., 2006). The Frisco-BL colony of human body lice was originally collected from nine homeless individuals in San Francisco (CA, USA) by Dr. Jane Koehler (University of California San Francisco Medical Center, San Francisco, CA, USA) in December 2008. Both colonies have been maintained by the Clark Laboratory at the University of Massachusetts–Amherst on human blood using the same in vitro rearing system (Yoon et al., 2006) under environmental conditions of 30 °C, 70% relative humidity, and an LD 16:8 hr photoperiod in rearing chambers (University of Massachusetts–Amherst Institutional Review Board approval no. E1404/001-002).

**Figure 1:**
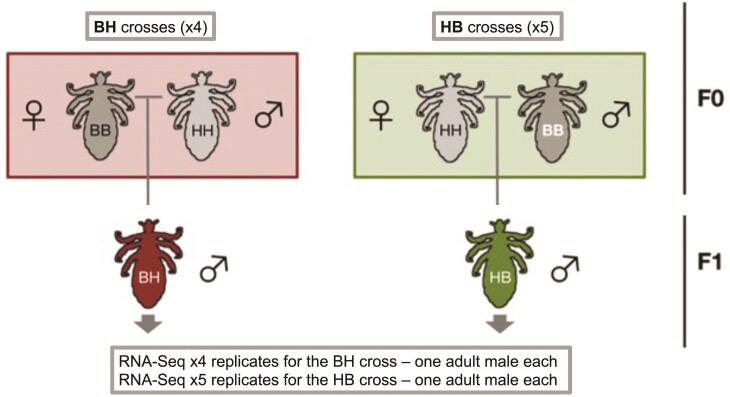
Schematic overview of the reciprocal cross design. Four individual male offspring were used for RNA-Seq from the BH (body lice female x head lice male) cross, creating four individual RNA-Seq libraries. Five individuals were used from the HB (head lice female x body lice male) cross for five RNA-Seq libraries.

Parental generations (F0) were established by isolating six sexually immature third-instar males and females from both colonies. F0 males from each ecotype were kept in common cages until sexual maturity, while F0 females were transferred to individual cages. Since sexually mature louse females can lay unfertilized eggs that do not hatch (Bacot, 1917), matings were delayed until females laid a first batch of eggs to confirm virginity. After 1-2 days, these eggs were removed from female cages to be incubated for a minimum of 10 days and then an F0 male from the other ecotype was introduced. Females were allowed to lay eggs for 15 days before the removal of the mating pair. In total, five head louse (HH) female x body louse (BB) male crosses (HB1, HB2, HB3, HB5, and HB6) and four BB female x HH male crosses (BH1, BH4, BH5, and BH6) were successful (Figure 1). Adult F1 males were collected in RNAlater after a 24-hr period of starvation. They were collected after their final molt, which occurs between 7 and 10 days of age (Takano-lee et al., 2003). In addition, to generate genomic and transcriptomic data from HH and BB ecotypes, adult individuals were directly isolated from the colonies (only males for RNA-seq, from both sexes for DNA-seq) and collected in RNAlater.

### RNA and DNA extraction and sequencing

RNA was extracted from a single F1 male per cross. Males were removed from RNAlater, washed twice in ice-cold 1X PBS, and ground in 400 μl of TRIzol (Invitrogen). Total RNA samples were isolated with a PureLink RNA purification kit (Thermo Fisher Scientific, USA), purified with RNA Clean & ConcentratorTM-5 (Zymo Research, USA), and validated using the Bioanalyzer RNA 6000 Nano kit (Agilent). TruSeq-stranded mRNA-seq libraries were generated by Edinburgh Genomics (UK) and sequenced on the Illumina NovaSeq platform (S2 flowcell, 50-bp paired-end reads). The depth of sequencing was ranged from 72,316,385 to 86,161,600 reads.

In addition to F1 RNA-seq, we also generated two replicates of RNA-seq data from pools of 10 adult BB and HH males from the same strains. Both HH and one of the BB samples were sequenced on the Illumina HiSeq 4000 (75 bp paired-end), generating between 59,734,668 and 82,349,787 reads per sample. The second BB sample was sequenced with the F1 RNA-seq, generating 66,095,517 reads. For whole-genome resequencing of parental strains, DNA was extracted using a DNeasy Blood & Tissue kit (Qiagen, The Netherlands) from pools of 10 adult individuals. TruSeq DNA Nano gel-free libraries (350 bp insert) and sequencing on the Illumina HiSeqX (150 bp paired-end) were performed by Edinburgh Genomics. Genome coverage was 18.8X for the body louse sample and 18.6X for the head louse sample.

### Generation of N-masked genomes

Whole-genome resequencing data were quality checked using fastqc v.0.11.5 (Andrews, 2010) and aligned to the reference genome (JCVI_LOUSE_1.0, Kirkness et al., 2010) using bowtie2 v.2.3.5.1 (Langmead & Salzberg, 2013) in *–sensitive* mode. The alignment rate for the body louse sample was 84.43%, and for the head lice sample, it was 80.52% of reads mapped. Data were deduplicated, and read groups added using picard v.2.6.0 (BroadInstitute, 2019). Less than 1% of all reads were marked as duplicates. SNPs were called using freebayes v.1.1.0 (Garrison & Marth, 2012) and filtered using vcftools v0.1.14 (Danecek et al., 2011), requiring a minimum depth of 10 reads and a minimum quality score of 20. Bedtools v.2.28.0 (Quinlan & Hall, 2010) was then used to identify homozygous alternative SNPs unique to each ecotype, this resulted in 204,411 SNPs unique to HH and 156,114 SNPs unique to BB. An N-masked genome was then created using all of these SNPs via the *maskfasta* command from Bedtools v.2.28.0 (Quinlan & Hall, 2010). The creation of an N-masked genome avoids later mapping bias to the reference allele.

### Identification of parent-of-origin and ecotype-of-origin expression

Male hybrid F1 offspring RNA-Seq data were quality checked using fastqc v.0.11.5 (Andrews, 2010) and trimmed with CutAdapt v.1.11 (Martin, 2011). Reads were aligned to the N-masked genome created above using RSEM v.1.3.0 (Li & Dewey, 2011), implementing STAR v.2.7.1 (Dobin et al., 2013) with standard parameters. Alignment rates were between 74% and 89% of reads mapped. Bam-readcount v.1.0.1 (https://github.com/genome/bam-readcount) was then used to count the number of reads that map to each of the four nucleotide bases for all positions that contain a homozygous alternative SNP in either head or body lice, identified above from the whole-genome resequencing data. Count data for the parental nucleotide at each of these positions were then extracted, and all positions that fell within a gene were annotated with a gene id using a custom R script. Only genes that had an SNP covered by a minimum of 10 reads in at least two replicates per cross direction were kept for further analysis. This left a final list of 481 genes suitable for testing for parent-of-origin expression effects. Genes showing parent-of-origin or ecotype-of-origin expression were determined using a logistic regression model in R, with a quasibionimal distribution to account for overdispersion. Correction for multiple testing was carried out using the Benjamini–Hochberg method (Benjamini & Hochberg, 1995). Genes were determined as showing parent-of-origin expression if the maternal/paternal allelic ratio (head/body lice allelic ratio for ecotype-of-origin expression) corrected *p*-value was <.05, and the parental expression proportion was >0.6 in both cross directions. An absolute cut-off of 0.6 was applied in line with previous research (e.g. de la Filia et al., 2021; Galbraith et al., 2016; Marshall et al., 2020; Smith et al., 2020) to minimize false positive calls. All custom scripts are available online, see Data and code availability.

### Differential gene expression between ecotypes

A differential gene expression analysis was carried out between the parental ecotypes of head (HH) and body (BB) lice. RNA-seq data were quality checked with fastqc v.0.11.5 (Andrews, 2010) and trimmed using CutAdapt v.1.11 (Martin, 2011). Reads were aligned to the N-masked genome created above, and transcript abundances were calculated using RSEM v.1.3.0 (Li & Dewey, 2011), implementing STAR v.2.7.1 (Dobin et al., 2013) with standard parameters. Alignment rates were between 76% and 90% of reads mapped. DESeq2 v1.28.1 (Love et al., 2014) was used to determine differentially expressed genes between ecotypes. A gene was considered differentially expressed if the corrected *p*-value was <.05 (adjusted for multiple testing using the Benjamini–Hochberg procedure (Benjamini & Hochberg, 1995)), and the log2 fold-change was >1.5.

### Protein blast and GO annotation

A reciprocal protein blast was carried out to identify the genes involved in DNA methylation maintenance (DNMT1) and establishment (DNMT3a) using 321 insect DNMT1 gene sequences and 110 insect DNMT3a gene sequences from http://v2.insect-genome.com/Pcg, using blastp v2.2.3 (Camacho et al., 2009). DNA methylation appears to be present in *P. humanus* based on the CpG observed/expected proxy (Bewick et al., 2017). We also then examined this specifically within exons and introns of all genes following custom scripts (https://github.com/MooHoll/cpg_observed_expected), see Supplementary 2: Supplementary Figure S7.

Gene ontology (GO) terms were annotated for 4,606 genes of 9,830, with gene expression data using eggNOG-mapper v.2.0.0 with standard parameters, i.e., the taxonomic search is adjusted per query and therefore not restricted to a single taxon (Cantalapiedra et al., 2021). GO enrichment was carried out in R using GOstats v2.56.0 (Falcon & Gentleman, 2007), which implements a hypergeometric test with Benjamini–Hochberg correction for multiple testing (Benjamini & Hochberg, 1995). GO biological processes were classed as over represented if the corrected *p*-value was <.05. REVIGO (Supek et al., 2011) was then used to visualize GO terms. GO terms from maternally biased and ecotype-biased genes were tested for enrichment against all genes present in the hybrid RNA-seq data as a background set. GO terms from differentially expressed genes between pure ecotypes were tested for enrichment against all genes present in the pure RNA-seq data as a background set. *Drosophila* orthologs for the maternally biased genes were determined using VectorBase (Amos et al., 2022).

## Results

### Parent-of-origin expression in F1 hybrid offspring

**Figure 2:**
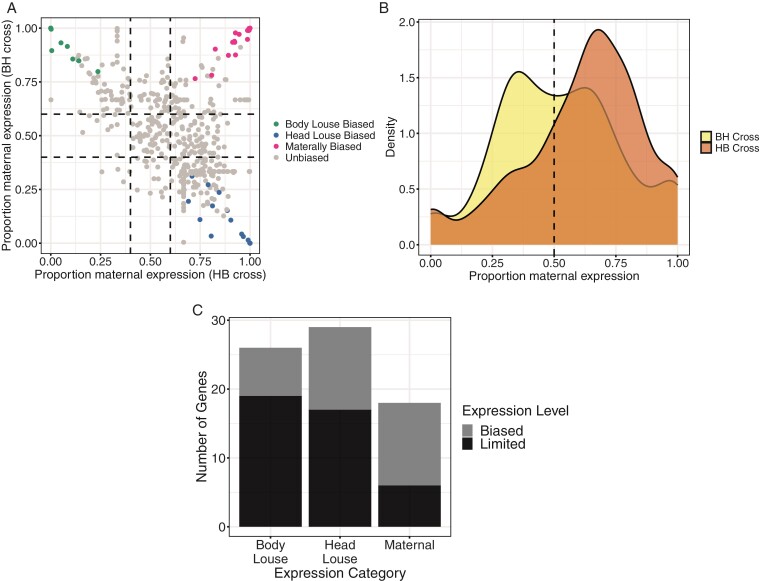
(A) Scatter plot showing the proportion of maternal expression for each cross direction for all genes tested (*n* = 481). Each dot is a gene. The black lines represent 0.4 and 0.6 expression proportions; these were used as a hard cut-off in addition to a significant *p*-value in order to call a gene biased. (B) Density plot showing the distribution of genes by maternal expression proportion for each cross direction (*n* = 481). BH = female body louse x male head louse, HB = female head louse x male body louse. (C) Staked bar plot showing the number of genes with expression bias in the reciprocal crosses. Biased genes are those with a significant maternal/paternal or head/body lice allelic ratio in both cross directions, corrected *p*-value <.05 and biased expression proposition >0.6. Limited genes meet the above criteria; however, they show complete silencing of one allele, i.e., expression is limited to either the maternal/head louse/body louse allele.

Males of most species with PGE tend to have eliminated or silenced paternal chromosomes within somatic tissue, meaning predominately maternal alleles are expressed (de la Filia et al., 2021; Herbette & Ross, 2023). Using reciprocal crosses of head and body lice, we were able to identify parent-of-origin expression in hybrid male offspring of *P. humanus* for 481 genes (Supplementary 1: Supplementary Table S1). Surprisingly, we find the majority of these are biparentally expressed, i.e., the paternal allele is not silenced (Figure 2A). However, there is a slight skew toward more maternal expression in both crosses generally (except for genes with head lice-specific bias, discussed below), Figure 2B. We also find 18 genes with significant maternal expression bias, of which six show complete silencing of the paternal allele (Figure 2C). None of these significant maternally biased genes are differentially expressed between pure head and body lice males.

A GO enrichment analysis of the maternally biased and maternally limited genes reveals multiple functions, specifically many associated with microtubule and actin filament organization (Supplementary 1: Supplementary Table S2; Supplementary 2: Supplementary Figure S1), with the GO terms “*meiotic spindle organization*” (GO:0000212), “*establishment of mitotic spindle orientation*” (GO:0000132), and “*mitotic spindle elongation*” (GO:0000022) specifically enriched. The segregation of maternal and paternal chromosomes during spermatogenesis is a key feature of PGE and, according to the genomic conflict hypothesis, is expected to be under maternal control. To explore this further, we identified *Drosophila* orthologs for the maternally biased genes, and we find the gene responsible for the enrichment of “*meiotic spindle organization*” (*xmap215*) is an ortholog of the *mini spindles* gene in *Drosophila* (FBgn0027948, Supplementary 1: Supplementary Table S3). This gene is involved in microtubule organization during both mitosis and meiosis (Brittle & Ohkura, 2005; Moon & Hazelrigg, 2004). The enriched GO terms, and the identification of *xmap215* as maternally biased, suggest a role for some of the maternally biased/limited genes in the segregation of the maternal and paternal chromosomes during spermatogenesis. However, it is worth noting that only around 43% of all annotated genes within the current *P. humanus* genome have associated with GO terms, as such we are missing functional information for some of the maternally biased/limited genes.

### Ecotype-specific expression in F1 hybrid offspring

In addition to identifying parent-of-origin gene expression, we were also able to identify ecotype-of-origin expression in the above 481 genes (Supplementary 1: Supplementary Table S1). We find 26 genes show higher expression from the body louse allele compared with the head louse allele in the hybrid male offspring, of which 19 show complete silencing of the head louse allele (Figure 2A and C). We see a similar number of genes showing head louse allele expression bias, 29 total, with 17 of these being completely silenced from the body louse allele (Figure 2A and C). Interestingly, we also see a pronounced skew generally for higher head louse allelic expression (Figure 2B).

A GO enrichment analysis of these genes shows that significant body louse biased/limited genes are involved in many cellular regulatory processes and metabolic processes, including “*regulation of meiotic cell cycle*” (GO:0051445) (Supplementary 1: Supplementary Table S2; Supplementary 2: Supplementary Figure S2), whereas significant head louse biased/limited genes are more enriched for developmental processes, including “*adult somatic muscle development*” (GO:0007527), “*oligodendrocyte development*” (GO:0014003), and “*cellular component assembly involved in morphogenesis*” (GO:0010927) (Supplementary 1: Supplementary Table S2; Supplementary 2: Supplementary Figure S3). As with above, some of the biased/limited genes do not have GO annotations, and so some functional information is missing.

### Differential gene expression between ecotypes

Given the large amount of ecotype-specific expression that we see in hybrid offspring, we carried out RNA-Seq on adult male head and body lice to see whether these ecotype-specific expression patterns in hybrids match the parental ecotype. Using RNA-Seq from the parental ecotypes, we have identified differentially expressed genes between head and body lice males. We find the majority of the variability within the data is between the two body lice replicates; however, 45% is explained by ecotype (Supplementary 2: Supplementary Figures S5 and S6). The variability within body lice is potentially explained by the use of different sequencing platforms for these samples (see Methods). Additional variability within sample groups will result in a higher frequency of false negative calls in terms of differentially expressed genes between ecotypes. We are therefore confident in the genes we identify below. While the majority of genes show similar expression levels between ecotypes (Supplementary 2: Supplementary Figure S5), we do find 47 differentially expressed genes (adjusted *p*-value <.05 and log2 fold-change >1.5, Supplementary 1: Supplementary Table S4). Significantly more of these are upregulated in body lice compared with head lice (X^2^ = 9.383, *df* = 1, *p*<0.01, Supplementary 2: Supplementary Figure S5), with two genes being limited to body lice (i.e., with zero expression in head lice, Supplementary 2: Supplementary Figure S5: PHUM135320—*hypothetical protein* and PHUM619270— *chloride intracellular channel*).

GO terms enriched for differentially expressed genes between ecotypes are involved in a variety of processes including “*adult feeding behaviour*” (GO:0008343) and “*chitin-based cuticle development*” (GO:0040003). Additionally, multiple GO terms associated with epigenetic processes are enriched, including “*DNA methylation on cytosine within a CG sequence*” (GO:0010424), “*DNA methylation-dependent heterochromatin assembly*” (GO:0006346), and “*histone H3-K27 acetylation*” (GO:0043974) (Supplementary 1: Supplementary Table S5, Supplementary 2: Supplementary Figure S4). Of the two genes with body louse limited expression, one has no GO annotations (PHUM619270) and the second (PHUM135320) is involved in wound healing (GO:0042060, GO:0009611) and response to stress (GO:0006950). Two of the most upregulated genes in head lice compared with body lice are involved in nervous system development (PHUM494820) and immune response (PHUM365700—*Defensin 1*).

After identifying these differentially expressed genes between ecotype, we checked to see whether the genes with ecotype-specific bias in hybrid males are also differentially expressed between pure head and body lice males. We find only one gene that shows both ecotype-specific expression in hybrid males and is also differentially expressed between pure ecotypes. *Programmed cell death protein* (PHUM549420) shows higher head lice allele expression in hybrid males, but higher expression in pure body lice males compared with pure head lice males.

Given that as part of this study we have generated new RNA-Seq libraries, we decided to see whether we could corroborate previous work, which identified differences in alternative splicing between head and body lice (Tovar-Corona et al., 2015). We used the IsoformSwitchAnalyzeR v.3.16 R package (Vitting-Seerup & Sandelin, 2019) to examine differentially alternatively spliced genes between ecotypes within our study. We did not identify any significant differentially alternative spliced genes between ecotypes. Therefore, we cannot corroborate the previous study. We believe that this is likely due to the lack of annotation of alternative transcripts within the current genome annotation, rather than ecotypes actually lacking differentially alternatively spliced genes. A larger dataset, generated specifically to address this question, in addition to an improved genome annotation and assembly is needed.

## Discussion

In this study, we used RNA-Seq from male hybrid offspring of head and body lice ecotypes of *P. humanus* to identify both parent-of-origin and ecotype-of-origin gene expression patterns. We find that hybrid offspring show mostly biparental expression of genes, meaning that the paternal chromosomes of males are not transcriptionally silent in *P. humanus*, contrary to what is seen in many other species with PGE. We find no genes with paternal expression bias; however, we do find several genes with maternal expression bias, some of which are involved in meiosis and chromosome segregation, which means that they could play a role in the elimination of the paternal chromosomes during spermatogenesis. We also find several genes that are expressed in an ecotype fashion, i.e., either the head/body louse allele is always more expressed in hybrid offspring compared with the other allele. We find these ecotype-specific expressed genes in hybrid offspring are mostly not differentially expressed between adults of the pure ecotypes. Finally, we have identified a potential role for epigenetic processes in ecotype differences, as indicated by differentially expressed genes between ecotypes being enriched for epigenetic-related GO terms.

### Transcriptionally active paternal chromosomes in males

The identification of full biparental gene expression in *P. humanus* across the entire genome is the first documented case in a species with PGE. Recent work in the mealybug *Planococcus citri*, which also displays PGE, used RNA sequencing to show almost no expression of the paternal genes in males (de la Filia et al., 2021). In de la Filia et al. (2021), a few genes did show some level of paternal expression, meaning they escape full silencing; however, this was less than 1%. In another scale insect clade (Ericoccidae) there is variability in the presence and size of a heavily condensed heterogametic body containing the paternal chromosome set and also variability in the degree of paternal gene expression between closely related species (Hodson et al., 2023). This finding suggests that patterns of male gene expression and ploidy might show substantial turnover within scale insects. This situation might also be the case in lice where PGE is found across all parasitic lice and one genus of booklice (*Liposcelis*) that is likely the sister clade to the parasitic lice. While all parasitic lice studied so far lack paternal chromosome heterochromatinization, *Liposcelis* species do exhibit paternal chromosome heterochromatinization (Hodson et al., 2017). It is currently not clear what drives transitions between biparental and uniparental (maternal) expression in males under PGE. The current leading hypothesis is that there is ongoing intragenomic conflict between the paternal and maternal genome over transmission in males. Paternally inherited alleles might be selected to escape elimination during spermatogenesis, while maternally inherited alleles could be selected to avoid this by transcriptional silencing of paternal alleles (Herrick & Seger, 1999; Ross et al., 2010). It is possible, however, that there are selective drivers such as sexual-antagonistic selection for ploidy, but these have yet to be explored in the context of PGE.

The lack of paternal genome silencing that we find might also have implications for sex determination in lice. Lice do not have sex chromosomes, and as such, males and females are genetically identical (Herbette & Ross, 2023). And unlike in other clades with PGE, there appears to be no sex difference in ploidy, resulting from either silencing or early elimination of paternal chromosomes. Sex determination in PGE taxa remains poorly understood, but has been hypothesized to be dependent on maternal factors deposited in eggs prior to fertilization (Herbette & Ross, 2023). Such a mechanism would therefore not depend on any genetic or ploidy differences between the sexes and is therefore compatible with our findings.

It is also worth noting that the biparental expression we have identified appears to be skewed toward higher expression of maternal alleles generally (with the exception of head lice biased expression). This is unlikely due to mapping bias to the reference genome as we employed strict criteria to avoid this. Additionally, the reference genome was created from a body lice sample. As such, any mapping bias would favor reads from body lice alleles, not in a parent-of-origin-specific way. We speculate that the general maternal bias may be caused by paternal silencing in some tissues, but not others. Another possibility is that the mechanisms to silence paternal alleles are not consistently successful across the paternal chromosomes. This could lead to reduced levels of paternal expression without complete silencing. The application of single-cell RNA sequencing to this system would help to determine the consistency of maternal allele expression bias across tissues.

In addition to mostly biparental gene expression, we have also identified a number of genes that show significant maternal expression bias or completely limited expression from the maternal copy. This is reminiscent of genomic imprinting, the parent-of-origin specific expression of certain alleles, common in mammals and flowering plants, but for which the existence outside of these clades remains debated. Potentially imprinted genes have been identified in other insect species that do not show PGE. Both the honeybee, *Apis mellifera*, and the bumblebee, *Bombus terrestris*, have been shown to display patent-of-origin gene expression (Galbraith et al., 2016; Marshall et al., 2020). However, there is no evidence of parent-of-origin gene expression in many other species including the jewel wasp, *Nasonia vitripennis* (Olney et al., 2021; Wang et al., 2016). To our knowledge, our finding in lice is therefore one of the clearest examples of genomic imprinting found in arthropods, and the first outside of the Hymenoptera.

The presence of a fraction of imprinted genes in human lice was predicted by earlier theoretical analyses: PGE is akin to whole-genome meiotic drive of the maternal genome and is therefore expected to be controlled by maternally derived alleles. In the absence of somatic adaptations to prevent or reduce global expression of paternal alleles, individual maternally imprinted genes can be expected to be involved in reproductive functions under PGE, chiefly during spermatogenesis. Differential segregation of paternal and maternal chromosomes in meiosis is key to the evolution and maintenance of PGE. Lice display a highly modified achiasmatic meiosis, followed by several rounds of mitotic division at the end of which an asymmetrical division produces functional spermatids carrying the maternal genome, and degrading nuclei carrying the paternal genome. Differential segregation most likely occurs during the last of the series of mitotic divisions preceding the formation of active spermatids (de la Filia et al., 2018; McMeniman & Barker, 2006). This stage is a clear candidate for maternally controlled segregation and elimination of parental chromosomes. In this context, the identification of *xmap215* as maternally biased is particularly intriguing. The *Drosophila* ortholog of this gene (*mini spindles*) has been shown to be involved in both stabilizing and destabilizing microtubles (Brittle & Ohkura, 2005), which could be important for the differential segregation of the maternal and paternal chromosomes. While usually the mini spindle protein localizes at centromeres (Lee et al., 2001), it can bind to noncentromere regions (Deng et al., 2021). Given that *P. humanus* is holocentric (Bressa et al., 2015), this adds to the possibility for a role of *xmap215* in PGE. While this is purely speculative, the identification of *xmap215* as a possible candidate for the regulation of PGE during spermanogenesis enables future immunoflourescence studies using xmap215 probes to identify chromosome binding sites and determine if these differ between maternal and paternal chromosomes. Functional studies using RNAi to knockdown expression would also allow the examination of its role in PGE.

Another expected category of maternally expressed genes under PGE are those involved in epigenetic tagging and expression control of paternal alleles. In mealybug and sciarid flies, paternal chromosomes display differential patterns of epigenetic marks, such as histone modifications (Bongiorni & Prantera, 2003; Escribá et al., 2011; Goday & Ruiz, 2002; Khosla et al., 2006). While the GO enrichment analysis of the maternally biased genes identified here does not show any functions related to epigenetic processes, it is worth noting we were only able to assign GO terms to <50% of these genes. Therefore, many functions of these genes remain unknown. Nevertheless, *P. humanus* does have a functioning DNA methylation system. DNA methylation appears to be involved in the silencing of paternal chromosomes in male mealybugs (although it is not clear if this is a cause or consequence of PGE) (Bain et al., 2021). An improved genome annotation for *P. humanus*, in addition to the identification of epigenetic differences between the maternal and paternal chromosomes, would aid in the identification of the role of maternally mediated epigenetic labeling in this system.

Together these findings provide the required groundwork for the identification of specific genes that may function to direct PGE within *P. humanus*. Future work utilizing low-input methods, such as single-cell RNA sequencing, would allow screening for maternally expressed genes specifically in male gonad tissue where PGE takes place.

### Male ploidy and the evolution of resistance

The rate at which species respond to natural selection determines how fast they are able to adapt to changing environments. This process is relevant in lice in order to predict the evolution of resistance to newly introduced pediculicides used to treat louse infestations. The rate of adaptation is likely dependent on a species’ reproductive genetics. There has been some suggestion previously that the hemizygous expression in males under haplodiploidy and PGE could contribute to an increased rate of adaptation as rare recessive beneficial mutations are exposed to selection (Klein et al., 2021). However, we show that this is unlikely to occur in lice as males are fully diploid. Instead, response to selection in males is likely impeded as males express a diploid set of genes but only pass on a haploid complement. Recent theoretical work shows that this leads to a reduction of the invasion probability of new alleles, particularly for male-function genes (Klein et al., 2021). This suggests that any control strategy aimed at males might be more robust and longer lasting than previously thought, due to a potentially slower speed at which resistance is able to evolve.

### Ecotype-specific gene expression

In addition to maternally biased genes, we were also able to identify genes, which show ecotype-specific expression. We find similar numbers of genes showing significant head louse expression bias to those showing body louse specific bias, with a large proportion of both having limited (i.e., monoallelic) expression. We also find higher expression generally from the head louse allele across genes. These asymmetric expression patterns are probably caused by cis-acting regulatory variants favoring the expression of one allele in both reciprocal crosses (Pollard et al., 2008; Wang & Clark, 2014). A previous study using reciprocal crosses of two related wasp species also identified a large number of species-specific expression bias in hybrid offspring (Wang et al., 2016). However, reciprocal crosses within species of honeybee (Galbraith et al., 2016) and bumblebee (Marshall et al., 2020), revealed little to no line-specific expression. The species status of head and body lice has been long debated, but mounting evidence supports that their phenotypic differences are due to ecological factors and even their subspecies status has been questioned (Light et al., 2008). Currently, they are considered different ecotypes of *P. humanus*: Body lice are believed to emerge regularly from head louse populations by colonizing new breeding grounds in human clothes (Li et al., 2010). However, the identification of genes with ecotype-specific expression in hybrids is suggestive of a divergence between the two ecotypes.

We used genetic differences (i.e., SNPs) between ecotypes to identify parent/ecotype-specific gene expression. Previous work has identified very few genetic differences between head and body lice (Li et al., 2010). The low levels of divergence between the ecotypes meant that we were only able to assess parent/ecotype-specific expression in 481/10,992 annotated genes due to the low occurrence of differentiating SNPs between ecotypes within gene bodies. Laboratory crosses of *P. humanus* like those presented in this article are extremely challenging, particularly for headlice, with the line used being the only lab-reared line currently in existence (Yoon et al., 2006). Therefore, presently, it seems unlikely that further crosses can be conducted between lines that are more divergent than those used in this study. While there appear to be very few genetic differences between ecotypes, differences in alternatively spliced transcripts have been observed (Tovar-Corona et al., 2015). We did not have the resolution to examine differences in alternative splicing in our study; however, future work to examine the possibility of parent/ecotype-specific alternative splicing could be fruitful in this system. We did, however, find a few differentially expressed genes between ecotypes. Previous evaluations of differentially expressed genes between pools of head and body lice from all developmental stages (instead of adult males only) are available, ranging from as low as 14 genes (Olds et al., 2012) to 552 genes (Previte et al., 2014). One of the most differentially expressed genes in our study, which shows strong upregulation in head compared with body lice (or downregulation in body lice compared with head lice), is the immunity gene *Defensin 1*, part of the Toll pathway. This is consistent with previous studies (Kim et al., 2012) and is of interest given the different abilities of the two ecotypes to vector the bacterial pathogen *Bartonella quintana*. We also found that differentially expressed genes are enriched for epigenetic-related processes. DNA methylation has previously been implicated in alternative splicing in the honeybee (Flores et al., 2012), although its functional role in alternative splicing is debated (Oldroyd & Yagound, 2021; Duncan et al., 2022). Epigenetic differences between ecotypes could explain the differences in life history and also the lack of need for genetic differentiation. Additionally, given that PGE is epigenetically mediated in other systems, and given that our previous work has shown differences in patterns of paternal chromosome elimination between the two ecotypes (de la Filia et al., 2018), future work focusing on epigenetic differences between head and body lice and maternal/paternal chromosomes in males would be a promising avenue to better understand these systems.

## Conclusion

We have shown that adult males of *P. humanus* display biparental gene expression, which constitutes the first known case of a species with PGE in which genetic activity of paternal chromosomes in the soma is not affected by embryonic silencing or (partial or complete) elimination. The absence of adaptations to silence the paternal genome as a whole in the human louse can facilitate unmasking genes involved in PGE (either enforcing it when maternally imprinted or combating elimination when paternally imprinted), most specifically during spermatogenesis. For example, the identification of the maternally biased *xmap215* gene, which might play a role in chromosome behavior during meiosis, lends itself to future functional studies of the mechanisms of PGE. Finally, the identification of ecotype-specific expression in hybrids of head and body lice, in the context of low genetic diversity, is suggestive of the role of epigenetic processes in ecotype differences.

## Supplementary Material

qrae003_suppl_Supplementary_Figures

qrae003_suppl_Supplementary_Material

## Data Availability

Data have been deposited in GenBank under NCBI BioProject: PRJNA968062. All code is available at https://github.com/MooHoll/head_and_body_lice_imp.
