# Supplementary material for: Lack of paternal silencing and ecotype-specific expression in head and body lice hybrids
Source: Evol Lett. 2024 Feb 6;8(3):455–65. doi: 10.1093/evlett/qrae003 (PMC11134467; doi:10.1093/evlett/qrae003)
Supplement: qrae003_suppl_Supplementary_Figures [file qrae003_suppl_supplementary_figures.pdf]

Supplementary 2: additional figures

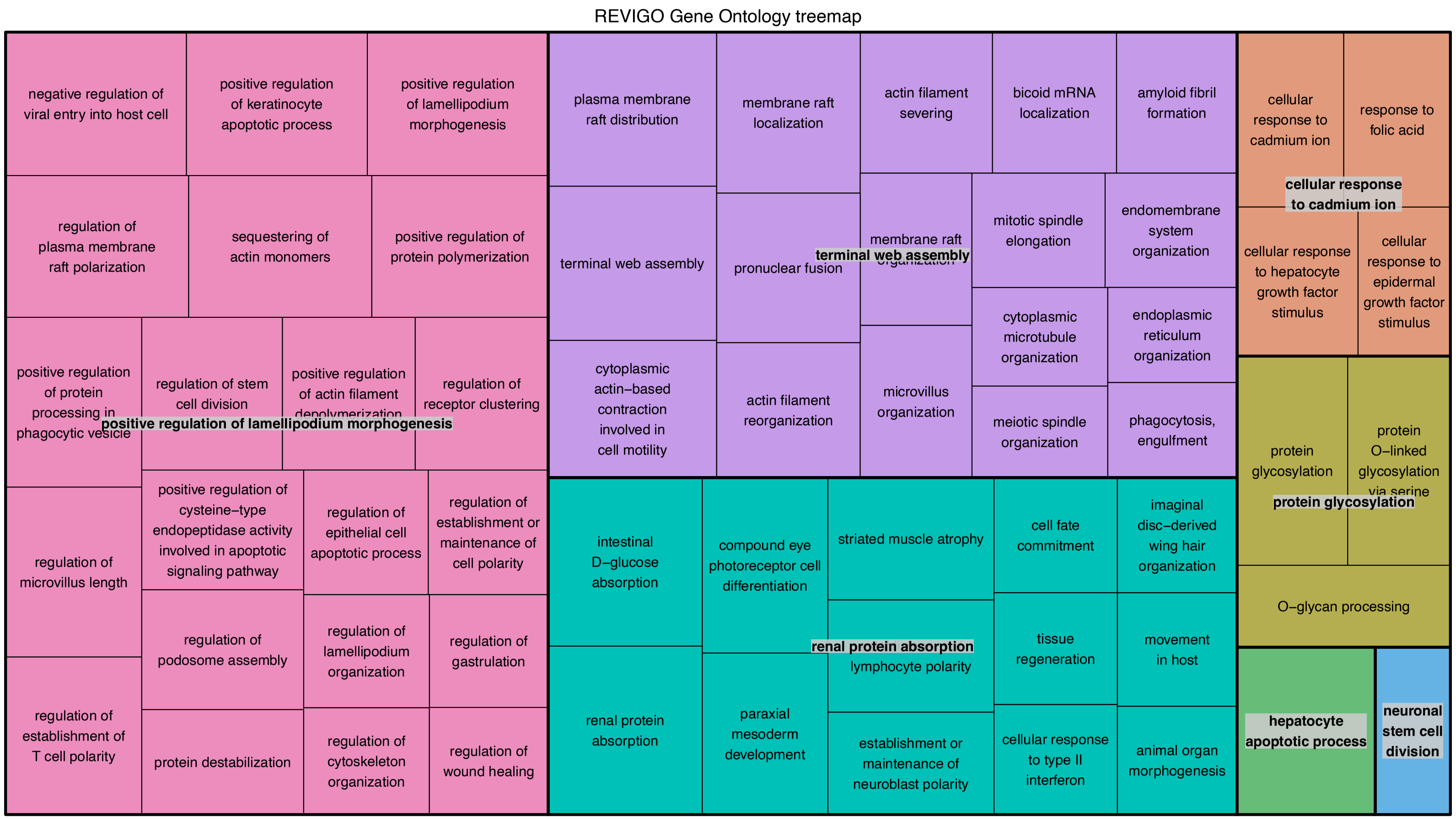

Figure S1: Treemap visualisation of the enriched GO terms for maternally biased genes. Each colour represents a specific cluster of related GO-terms and the size of each rectangle represents the size of the p-value, i.e. a larger rectangle is a smaller p-value.

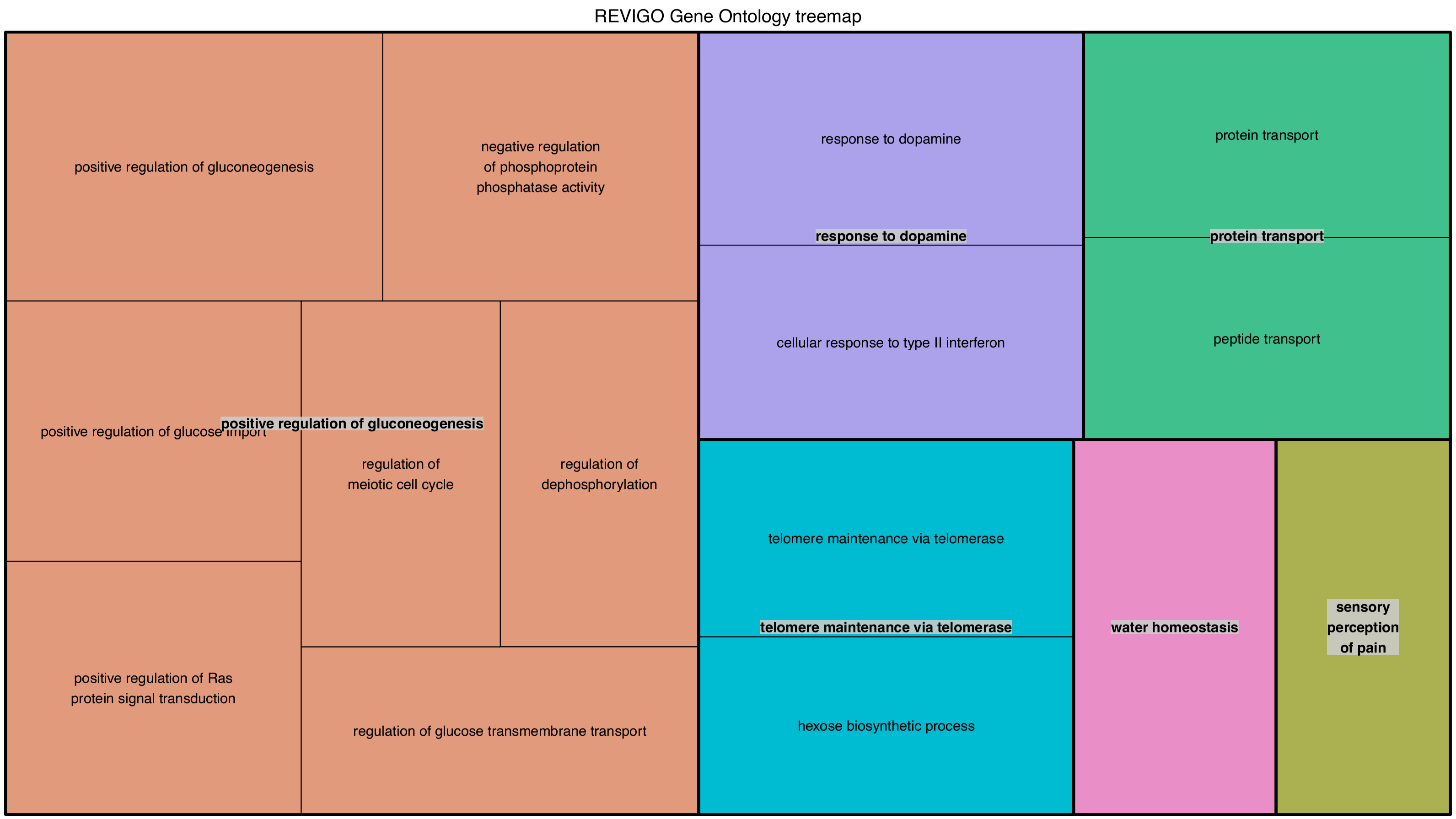

Figure S2: Treemap visualisation of the enriched GO terms for body louse biased genes. Each colour represents a specific cluster of related GO-terms and the size of each rectangle represents the size of the p-value, i.e. a larger rectangle is a smaller p-value.

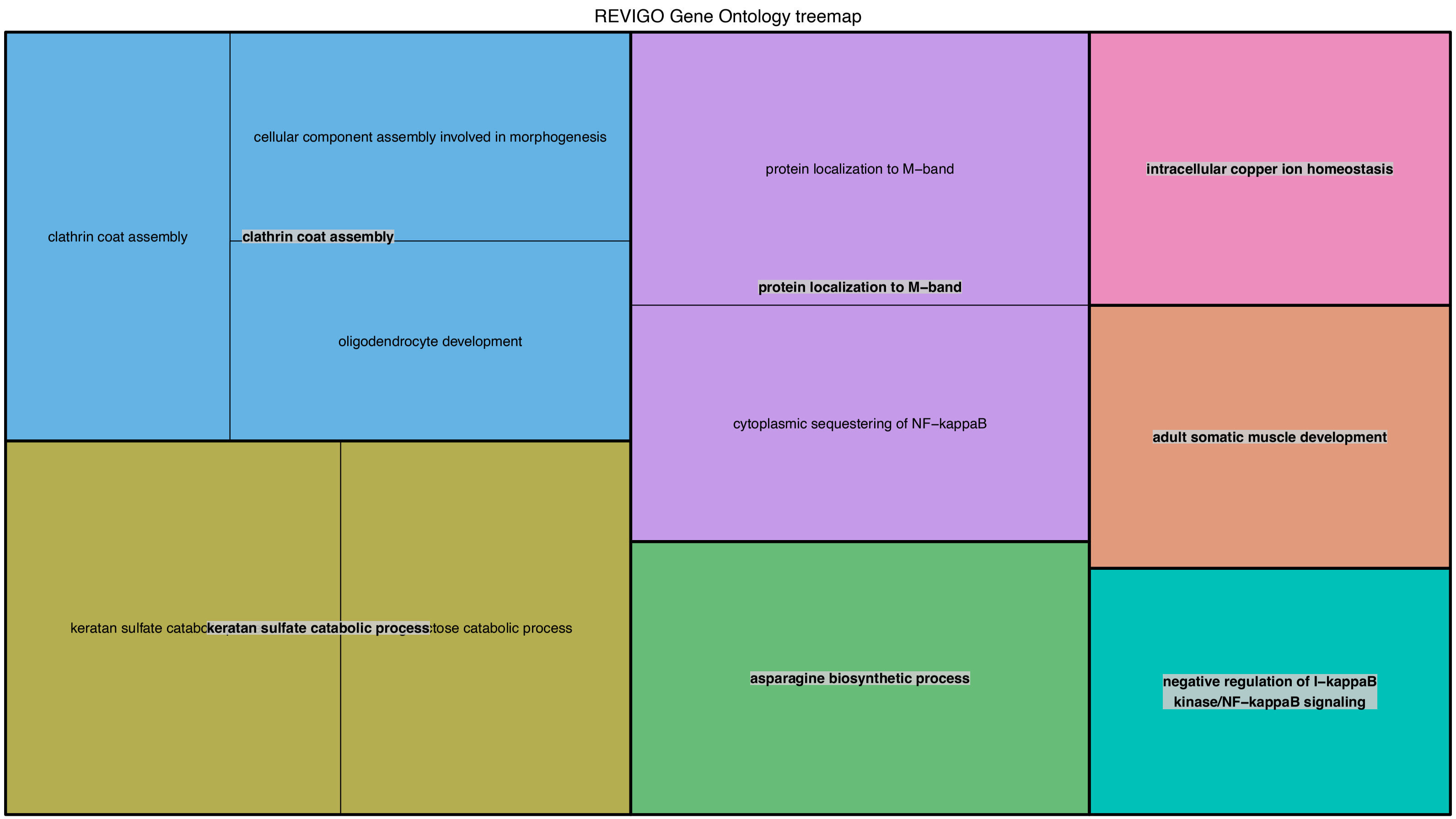

Figure S3: Treemap visualisation of the enriched GO terms for head louse biased genes. Each colour represents a specific cluster of related GO-terms and the size of each rectangle represents the size of the p-value, i.e. a larger rectangle is a smaller p-value.

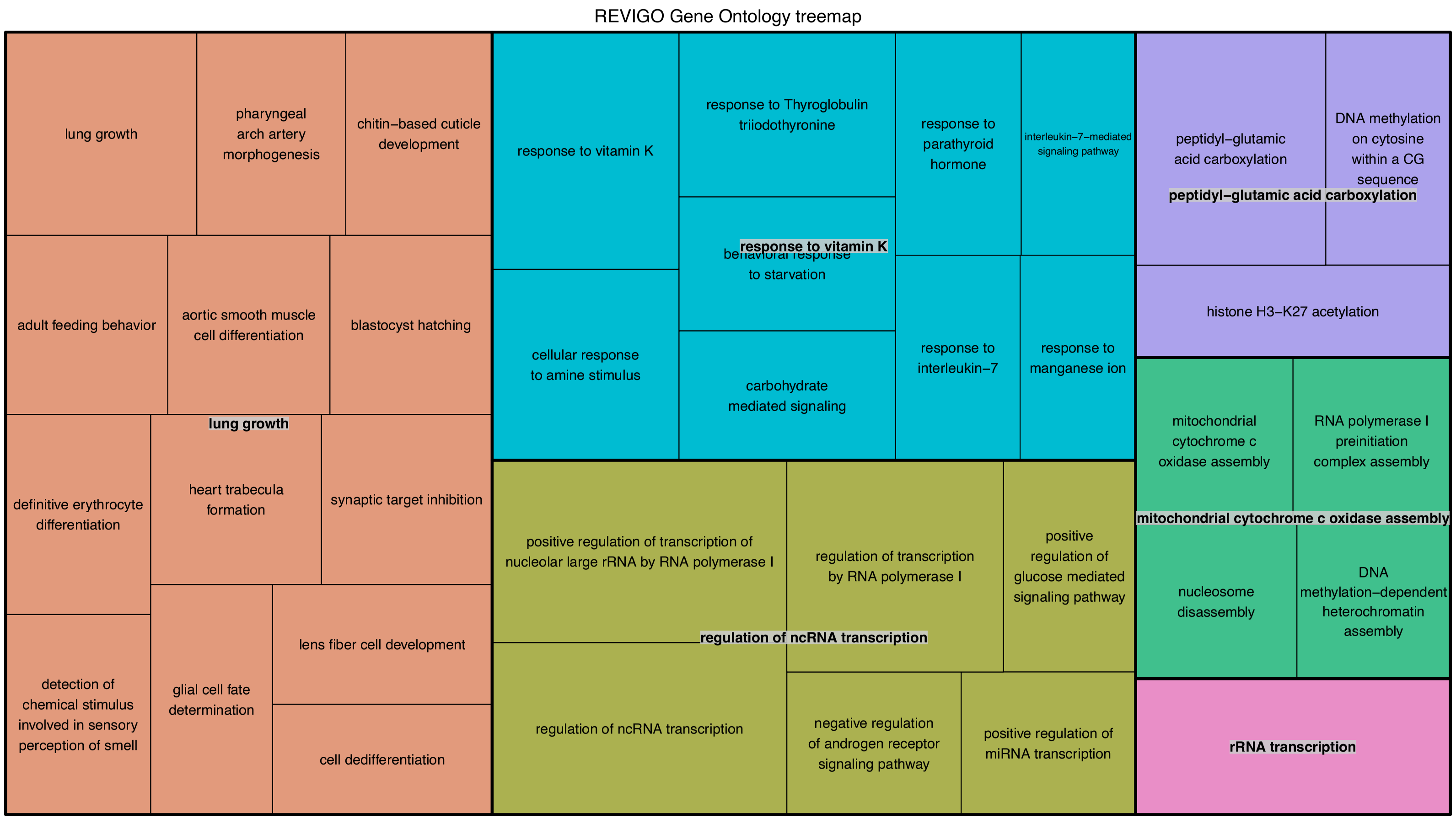

Figure S4: Treemap visualisation of the enriched GO terms for differentially expressed genes between ecotypes. Each colour represents a specific cluster of related GO-terms and the size of each rectangle represents the size of the p-value, i.e. a larger rectangle is a smaller p-value.

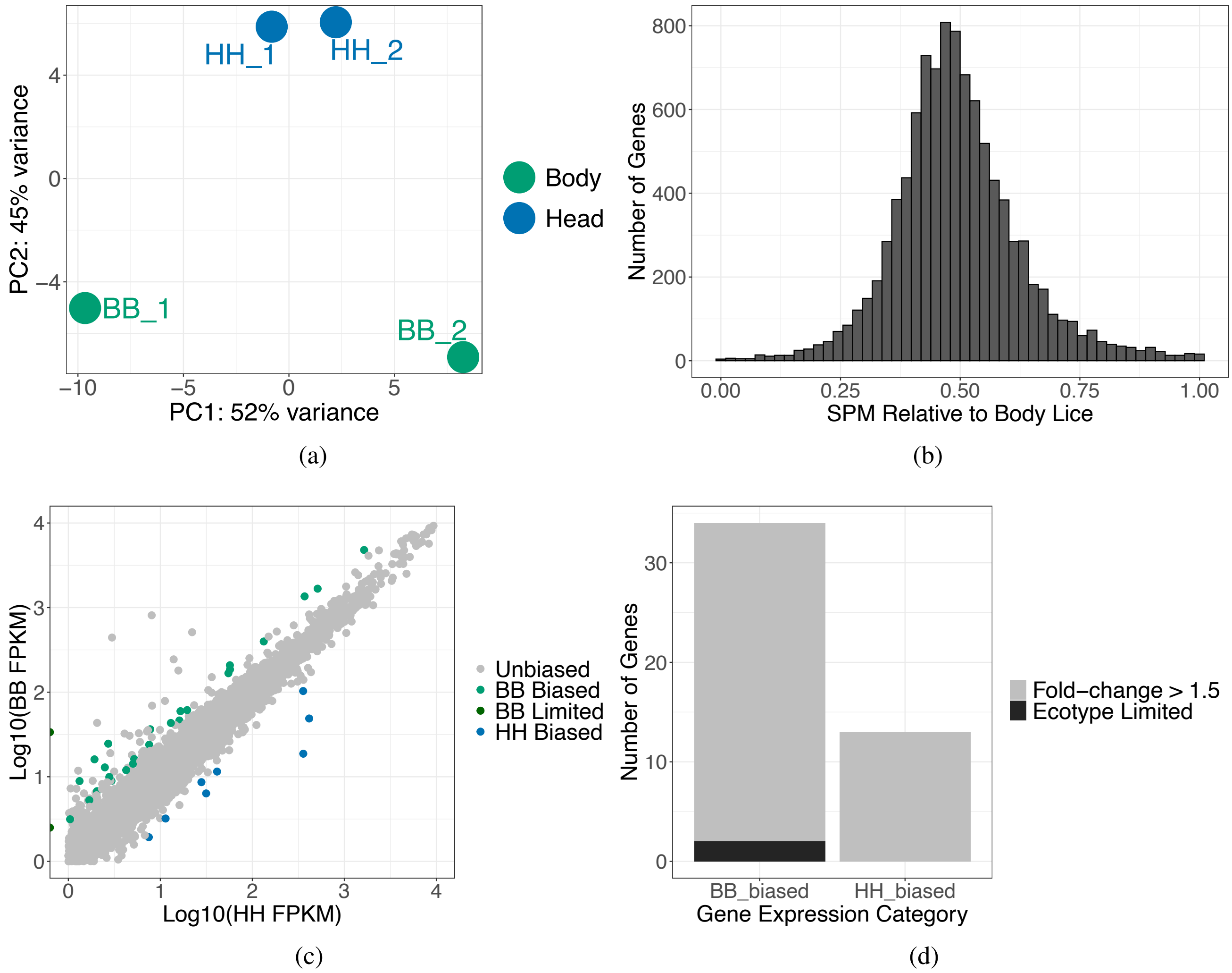

Figure S5: (a) PCA plot based on the expression of all genes in the head and body lice RNA-Seq (n = 9,830). (b) Histogram of the SPM (measure of specificity (Xiao *et al.*, 2010), calculated as body lice FPKM (Fragments Per Kilobase of transcript per Million mapped read) squared divided by body lice FPKM squared plus head lice FPKM squared) per gene (n = 9,830) showing a large number of genes are equally expressed between ecotypes. (c) Scatter plot of the log10 fragments per kilobase of transcript per million mapped reads (FPKM) of all genes (n = 9, 830). Significantly differentially expressed genes (corrected p-value <0.05 and log2 fold-change >1.5) are coloured by ecotype and level of differential expression, unbiased genes are shown in grey. (d) Stacked bar plot showing the number of ecotype-biased genes. Ecotype-limited genes referring to those with zero expression in one ecotype.

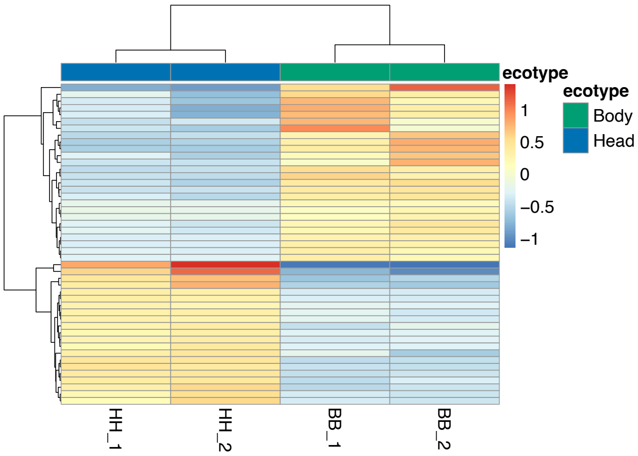

(a)

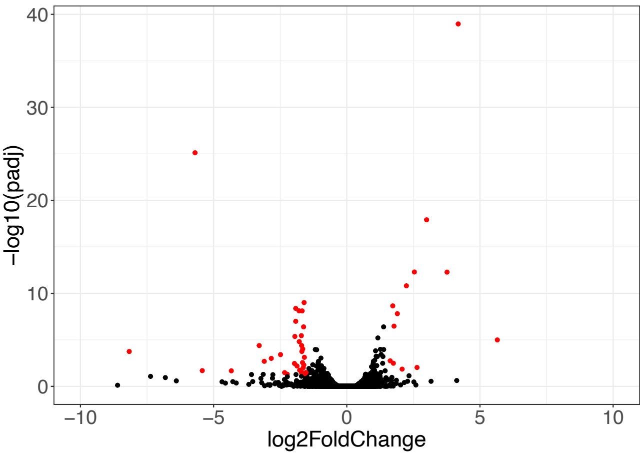

(b)

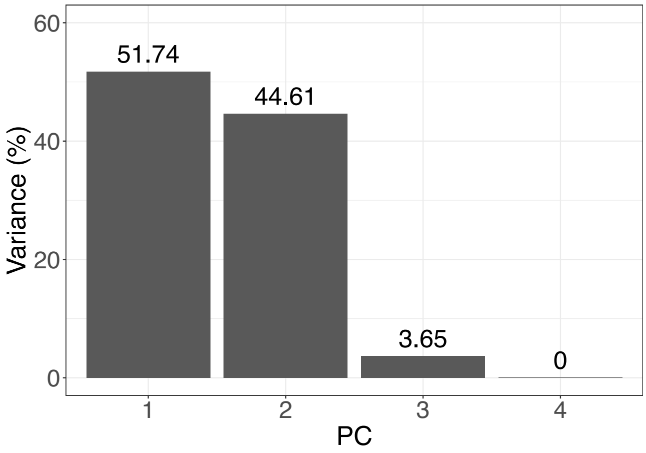

(c)

Figure S6: (a) Heatmap showing the top differentially expressed genes between head and body lice ecotypes. (b) Volcano plot showing significantly differentially expressed genes in red. (c) Scree plot showing the percentage variance captured by each principle component (PC) for the pure head and body lice RNA-Seq. The numbers above each bar show the percentage variance to two decimal places.

Given the genetic similarity between ecotypes, and given that our go term analysis highlighted enrichment for several epigenetic pathways in the differential gene expression analysis. We therefore decided to further explore the potential involvement of epigenetic processes in mediating ecotype differences. We identified genes involved in DNA methylation maintenance and establishment, DNMT1 and DNMT3a in *P. humanus*. We find similar levels of expression for both genes between ecotypes (supplementary 2: Fig.S7, DNMT1 (PHUM556380) adjusted p-val = 0.99, DNMT3a (PHUM041250) adjusted p-val = 0.95). Previous research has suggested DNA methylation is present in *P. humanus*, based on the the CpG observed/expected distribution (Bewick *et al.*, 2017). We checked this distribution in exons and introns and find DNA methylation is likely enriched in exons in *P. humanus* (Fig.S7), as is seen across arthropods (Lewis *et al.*, 2020).

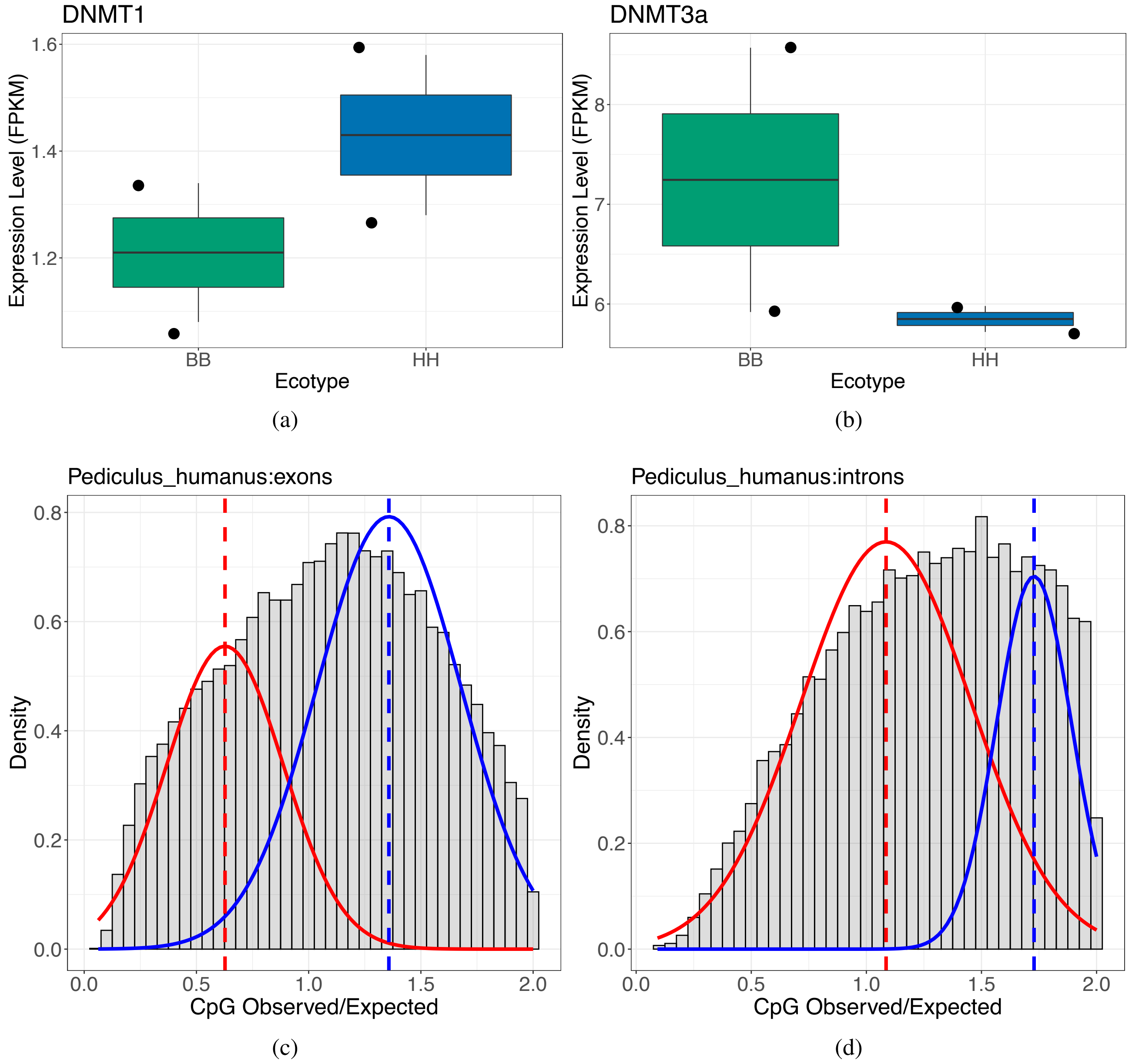

Figure S7: (a and b) Boxplots of expression levels of two DNMT genes. BB refers to body lice and HH refers to head lice. Each dot represents a single RNA-Seq library. (c) CpG observed/expected distribution for all exons in the *P. humanus* genome and for introns (d). CpG o/e is the ratio of the observed number of CpG sites in a given region compared to the expected number of CpG sites, given the GC content of that region. A depletion of CpG sites (CpG o/e < 1) is indicative of DNA methylation presence as DNA methylation degrades CpG sites over time due to the increased deamination of cytosines to thymines. Species with DNA methylation tend to show two CpG o/e peaks, one around 0.5 and another around 1.0, representing methylated and unmethylated regions.
